# Reduced Prevalence of Measles Antibodies in a Cohort of Brazilian Children under 15 Years of Age

**DOI:** 10.3390/vaccines10101570

**Published:** 2022-09-20

**Authors:** Cassia Fernanda Estofolete, Flora de Andrade Gandolfi, Bruno Henrique de Aguiar Milhim, Gislaine Celestino Dutra da Silva, Fernanda Simões Dourado, Bárbara Ferreira dos Santos, Nikos Vasilakis, Maurício Lacerda Nogueira

**Affiliations:** 1 Laboratório de Pesquisas em Virologia, Faculdade de Medicina de São José do Rio Preto (FAMERP), São José do Rio Preto 15090-000, SP, Brazil; 2 Hospital de Base de São José do Rio Preto, São José do Rio Preto 15090-000, SP, Brazil; 3 Hospital da Criança e Maternidade de São José do Rio Preto, São José do Rio Preto 15091-240, SP, Brazil; 4 Department of Pathology, The University of Texas Medical Branch, Galveston, TX 77555, USA; 5 Department of Preventive Medicine and Population Health, The University of Texas Medical Branch, Galveston, TX 77555, USA; 6 Center for Vector-Borne and Zoonotic Diseases, The University of Texas Medical Branch, Galveston, TX 77555, USA; 7 Center for Biodefense and Emerging Infectious Diseases, The University of Texas Medical Branch, Galveston, TX 77555, USA; 8 Center for Tropical Diseases, The University of Texas Medical Branch, Galveston, TX 77555, USA; 9 Institute for Human Infection and Immunity, The University of Texas Medical Branch, Galveston, TX 77555, USA

**Keywords:** measles, immunization, outbreaks, seroprevalence

## Abstract

Measles is a highly contagious acute febrile disease responsible for sporadic but explosive outbreaks worldwide. Although it was considered eliminated from the Americas, the disease reemerged in 2017. This reemergence was linked to reduced immunization rates. Vaccination, early identification of cases, and blocking of the transmission chain are the most effective tools to combat measles, for which there is not a specific antiviral treatment. In this Brazilian prospective cross-sectional study, we investigated the prevalence of measles antibodies in children, a population vulnerable to significant morbidity and mortality from exposure to infection. Between December 2018 to November 2019, blood samples from 252 children were collected and assessed for the presence of measles-specific IgG antibodies and an overall prevalence of 75.8% was observed. We further stratified the cohort into age subgroups, none of which exhibited antibody presence levels above the herd immunity threshold of 94%. Moreover, the calculated number of secondary cases resulting from a case in any age group ranged from 3 to 4, demonstrating that even with partial vaccination coverage this disease is still concerning and highly transmissible. Despite global warnings about measles and previous efforts to combat the advance of the disease, data on antibody prevalence and vaccination coverage in several countries are still concerning and indicate that significant parts of the population could be affected. Seroprevalence studies like this one are important to highlight actual epidemiological conditions and emphasize the need for additional strategies to encourage immunization and avoid the potential impacts of measles, particularly in children who can be severely affected.

## 1. Introduction

Measles virus (MeV) is an enveloped single-stranded RNA of negative polarity virus belonging to the family *Paramyxoviridae*, genus *Morbillivirus*, causing an acute, highly contagious illness presenting with a broad clinical spectrum ranging from an acute febrile exanthematous syndrome, characterized by high fever, runny nose, cough, conjunctivitis, and generalized maculopapular rash to severe complications, such as encephalomyelitis and death [1,2]. 

**Figure 1:**
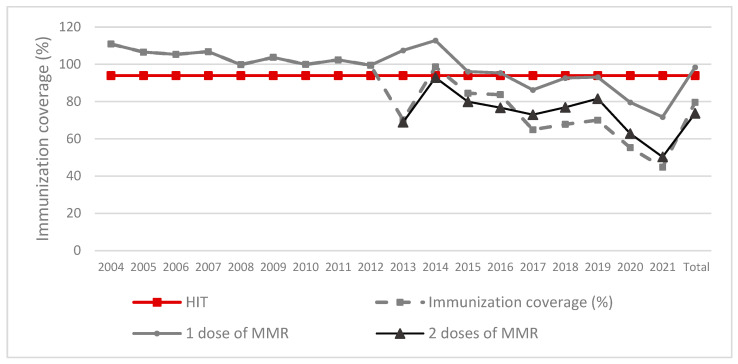
Annual measles vaccine coverage (%) in Brazil, 2004–2021, based on administered doses. Note: the 1 dose of MMR curve is higher than 100% because it documents the ratio of the number of doses applied and the total number of individuals in the population for each respective age group. Since the census is retrospective, a larger number of individuals are available for vaccination [7]. HIT: herd immunity threshold; MMR: measles, mumps, rubella.

To date, on a global, country, state, and community level the most effective tool of preventing and controlling measles outbreaks, as well as disease, is immunization. MeV is one of the most contagious diseases, with a basic reproduction number (R_0_) ranging from 12 to 18 [3] and can only be effectively controlled by vaccination coverage equal or higher than 95%, and early identification of cases [4]. The measles vaccine was introduced in Brazil in the 1960s and was only incorporated into the national immunization program in 1973, which significantly contributed to the decrease of infant mortality. Since 2004 in Brazil, the vaccine is delivered co-formulated with the rubella and mumps vaccines (MMR) [5] in a two-dose regimen, with the first dose given around 9 months to 15 months of age, and the second dose at 15 months to 6 years of age, with at least four weeks between doses. Protection against disease after a single dose is 93% (CI 39–100%), and 97% (CI 67–100%) following the second dose [6]. Although reporting of detected cases is compulsory in Brazil, which activates a cascade of control measures to contain spread of the disease, these measures have not halted the spread of this disease. Contributing factors include reduction of vaccine coverage, which between 2004 and 2021 has dropped well below the recommended coverage level to contain this disease (Figure 1) [7], as well as rising misconceptions on the risks of vaccination, religious and cultural beliefs, and immigration due to collapse of public health structures in the region [8,9]. 

Between 2013 and 2015, a resurgence of MeV was observed in Brazil with 1052 confirmed measles cases, resulting in a massive vaccination and immunization awareness campaign [10]. The success of the Brazilian vaccination policy implemented in 1992 with the introduction of the Plan for Measles Control and Elimination led to the eradication of MeV and certification in 2016 as an autochthonous measles transmission free country. However, autochthonous transmission of the virus re-emerged in 2018 with 10,346 confirmed cases in addition to imported cases, followed by 20,901, 8448, and 668 confirmed cases in 2019–2021 respectively [11,12]. In 2022, between epidemiological weeks 1 and 25, 41 cases were confirmed and another 453 are being investigated [13]. Such reemergence was facilitated by reduced coverage of herd immunity.

Our current knowledge of MeV contagiousness, its morbidity and mortality impact in the pediatric population, and access to immunization as the only route for its prevention and control reiterate the vital importance of comprehensive data on the immunological status of a given population in guiding public policy. In 2020, our study group noted reduced rates of measles antibodies in different age groups and the risk to which they were exposed [14]. In 2021, the incidence MeV antibody coefficient by age group reported to younger than 1 years of age 73.2 cases/100,000 inhabitants, followed by 14.71, 2.48, and 0.84 to 1–4, 5–9, and 10–14 years of age groups, respectively. The highest incidence coefficient was observed in the pediatric population, especially in the younger than 5 years of age group (27.34 cases/100,000 inhabitants) [15], suggesting that the disease continues to threaten the youngest pediatric population, who are the main target group of immunization programs. Designed to inform public health authorities and decision makers for implementation of sustainable vaccination plans aimed at preventing future outbreaks, our study presents a detailed evaluation of the prevalence of MeV antibodies in a pediatric population in Sao Jose do Rio Preto, São Paulo State, a city with 469,000 inhabitants, of which 78,705 are younger than 15 years of age. 

## 2. Materials and Methods

This study leverages the resources of an ongoing arbovirus surveillance cohort. Arbovirus infection-suspected individuals are routinely investigated, as per standard policy guidelines established by local public health authorities. Anonymized samples collected from children up to 15 years of age and enrolled between December 2018 and November 2019, were submitted for laboratory investigation for immunoglobulin G (IgG)-specific measles antibodies using a commercial enzyme-linked immunosorbent assay (ELISA Euroimmun AG, Lubeck, Germany). The procedures were performed according to the manufacturer’s instructions, and the qualitative measurement of IgG measles antibodies was categorized based on index standard ratio (ISR) values: seronegative was defined as: ISR < 0.8; indeterminate results were defined as: ISR between 0.8 and 1.1; and seropositive was defined as ISR > 1.1. Indeterminate results were excluded from the final analysis. 

Antibody results were organized into subgroups according to children’s ages: (i) up to 1 year of age; (ii) 1 year of age to 2 years of age (first and second doses of measles vaccine); (iii) 2 years of age to 5 years of age; (iv) 5 years of age to less than 10 years of age; and (v) 10 years of age to less than 15 years of age. The study population includes children with varying vaccination statuses: the subgroup of children up to 1 year of age included individuals who had not yet been immunized; those in the 2–5 years of age group may have been immunized or still receiving the series; the 2–5 years of age group may have been fully immunized, and children older than 5 years old were expected to be fully vaccinated. Complementary data were collected for statistical analysis, including such variables as: sex, ethnicity, and presence of comorbidities (asthma, kidney disease, liver disease, hematologic diseases, seizures, cancer, immunosuppression (e.g., cancer therapy, HIV infection, primary immunosuppression disease, and autoimmune disorder, genetic syndromes)).

The chi-square test was used to compare the categorical variables of the serological groups; *p*-value < 0.05 was considered statistically significant. The sample number was calculated using EPI Info^TM^ (version 7, CDC, Atlanta, GA, USA) assuming an expected baseline of positive anti-measles IgG of 95%. The basic reproduction number (R_0_) of 15.3 (3) was considered to calculate the herd immunity threshold (h) by equation h = 1 − 1/R_0_ and the number of secondary cases (R_e_) in each age group if a single infectious individual had contact with the remaining susceptible individuals by R_e_ = R0 × (1 − Pi), Pi being the percentual of immunized individuals in each age group. 

## 3. Results

Between December 2018 and November 2019, 252 children were included in our cohort, and had blood samples examined to detect IgG-specific measles antibodies. The children were predominantly older than 10 years of age (35.3%), male (52.8%), Caucasian (93.9%), and without comorbidities such as asthma, kidney disease, liver disease, hematologic diseases, seizures, cancer, immunosuppression, or genetic syndrome (85.5%) (Table 1). The overall cohort prevalence of measles-specific IgG antibodies observed was 75.8% (191/252). No differences in gender, age, ethnicity, or comorbidities were observed when all serological age groups were analyzed (Table 1). When comorbidities were evaluated in detail for conditions related to immunosuppression, the frequency of children with measles antibodies did not differ, even among immunosuppressed individuals (*p* = 0.808). 

**Figure 2:**
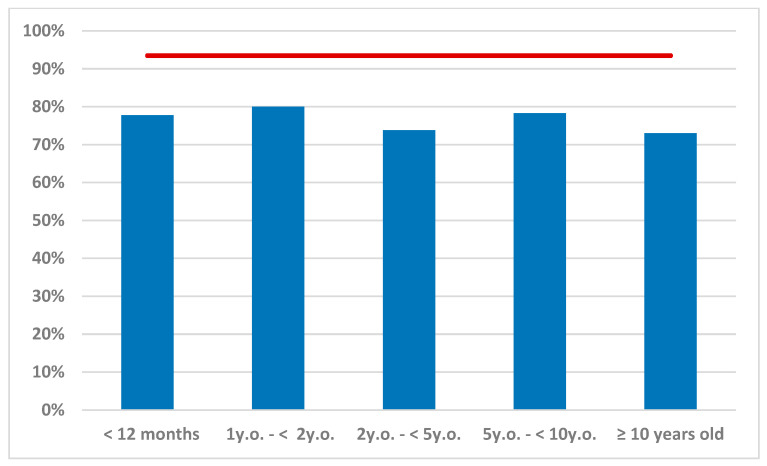
Estimated herd immunity threshold (red line) based on R_0_ = 15.3, and numbers of secondary cases in each age sub-cohort if a single infectious individual had contact with the remaining susceptible individuals (Re).

Considering a basic reproduction number of 15.3, the herd immunity threshold was estimated at 93.46%. The frequency of children who had measles-specific IgG antibodies did not exceed this limit in any age subgroup; the highest value (80%) was observed among children 1 to 2 years of age but was still far below the level to prevent the spread of this disease. The oldest subgroup (10 to 15 years of age) had the lowest percentage of children with measles antibodies (73%). The estimated number of secondary cases in each age subgroup if a single infectious individual had contact with the remaining susceptible individuals ranged between 3.1 and 4.1 (Figure 2).

## 4. Discussion

Measles may be considered ideal for eradication, since MeV has only one serotype, most cases are clinically identifiable, and the vaccine is freely available [1]. However, challenges related to this disease persist and are complex, involving political, technical, social, and economic factors [16]. Because immunization is the most effective tool to halt its spread, the low seroprevalence of measles antibodies in this population is an important warning of how far away we may be from eradication. This becomes even more concerning when considering pediatric populations that are most vulnerable to the clinical severity of infection [17].

In all age groups up to 15 years old, we found measles antibodies below the threshold level required to contain the spread of this disease. Historically, immunity developed by natural infection [18] or vaccination [17] is considered to be life-long. Our study population consisted of individuals in age ranges representing probable passive immunization through maternal-transferred antibodies (younger than 12 months old), as well as those in the process of vaccination (1 to 2 years age group) or who had already been immunized (greater than 2 years of age). High rates of measles antibodies were not found in the up-to-1-year age group, suggesting that passive immunity (via the placenta, delivery, or breastfeeding) was not acquired by these children and raising the possibility that their mothers also lacked antibodies due to lack of immunization. Further, lower titers and shorter duration of antibodies in newborns is associated with passive immunity acquired by maternal antibodies, which is predominantly by vaccination, rather than natural infection [19]. Because we did not obtain blood samples from the mothers of the participants under 1 year of age, this notion could not be confirmed. Furthermore, fewer children than expected were found to have measles antibodies in the age group (2 to 5 years of age) that should have recently completed the immunization process. 

Historical analysis of measles cases in Brazil since 2004 (when the current vaccine was effectively incorporated into the immunization program) demonstrates that the occurrence of cases follows the levels of vaccination coverage, with an increase in 2014 soon after coverage dropped below the herd immunity threshold [7,9]. In 2016, PAHO/WHO declared the Americas the first region in the world to be free of measles [10]. In fact, elimination was achieved in 2002 and by 2010 case numbers remained stable in the region, usually imported or related to imported cases. Some alerts related to such cases began to appear for some countries that experienced outbreaks between 2011 and 2015, highlighting the risk that measles-free status might be lost, as in Canada (803 cases in 2011), Ecuador (265 cases in 2011), and Brazil (27 cases in 2011) [20].

**Figure 3:**
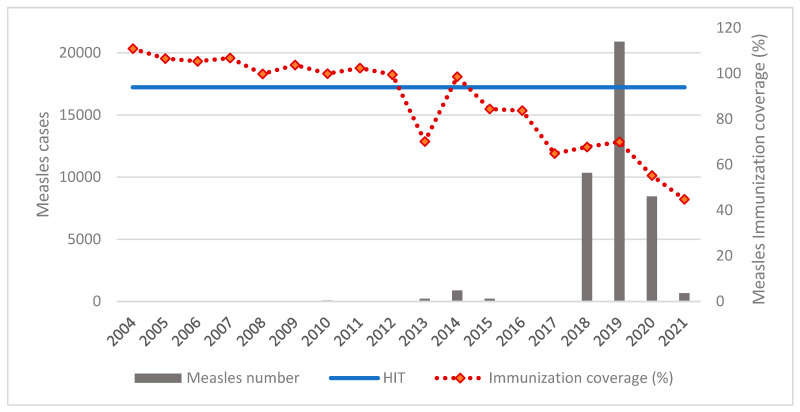
Relationship between measles case numbers and immunization coverage in Brazil, 2004–2021 [7,9]. HIT: herd immunity threshold.

In 2017, six countries in the Americas again had laboratory-confirmed cases of the disease: Antigua and Barbuda (1 case), Argentina (3 cases), Canada (45 cases), Guatemala (1 case), the United States (120 cases), and Venezuela (217 cases) [21]. At the end of 2018, the number of countries with confirmed cases of measles in the region reached twelve, including Brazil, Chile, Colombia, Ecuador, Mexico, and Peru [22]. Figure 3 depicts these epidemiological developments that followed the lowest percentage of measles vaccine coverage in the country, which occurred in 2017. 

Similar measles seronegativity data were also observed in children by other authors, including Gupta et al. [23], where 60% of children between 5 and 10 years of age in India lacked protective immunity against measles, and Sánchez-Alemán et al. [24], where 59% of Mexican children aged 6–13 years of age lacked MeV antibodies. Data reported by the WHO indicate that measles immunization coverage in the Americas has been approximately 69.2% over the past 20 years, with rates slightly higher after 2014; rates well below the threshold of herd immunity. Overall, this percentage was even lower (around 45%) during the 2000–2020 period and averaged roughly 66% between 2014 and 2020 [25].

## 5. Conclusions

Although the number of secondary cases after exposure in the different pediatric age groups is low compared to the R_0_ of measles, it remains significant, ranging from 3 to 4. Furthermore, none of the age groups was seen to be effectively protected. However, while the presence of measles antibodies is considered to offer life-long protection against infection, some studies have demonstrated that just their presence may not be sufficiently protective: instead, high titer levels may be required to realize their protective factor [26]. While our prospective study represented a cross-sectional and single-center study, the observed decreased prevalence of MeV-specific antibodies in the pediatric population warns of a severe epidemiological situation that has continued since the virus reemerged in 2017. Although measles vaccination is free and included in the Brazilian national immunization program, coverage is still lower than the recommended level, and if additional strategies to increase vaccination coverage are not instituted soon, we believe that significant measles outbreak waves will occur [27,28]. Accurate estimates of vaccination coverage, easy access to vaccines, and raising awareness of the importance of immunization through targeted communication drives are strategies that can contribute to expansion of vaccination coverage and help mitigate the disease impact of outbreaks. 

## Figures and Tables

**Table 1:** General characteristics of the 252 children assessed for IgG-specific measles antibodies.

	Global			IgG Negative		IgG Positive		
	N Total	N Positive	%	N positive	%	N Positive	%	*p*-Value
**Gender**								
Female	252	119	47.2%	24	20.2%	95	79.8%	0.157
Male	252	133	52.8%	37	27.8%	96	72.2%	
**Age Group**								
<12 months	252	18	7.1%	4	22.2%	14	77.8%	0.913
1 y.o.–<2 y.o.	252	20	7.9%	4	20.0%	16	80.0%	
2 y.o.–<5 y.o.	252	42	16.7%	11	26.2%	31	73.8%	
5 y.o.–<10 y.o.	252	83	32.9%	18	21.7%	65	78.3%	
≥10 years old	252	89	35.3%	24	27.0%	65	73.0%	
**Ethnicity**								
Caucasian	246	231	93.9%	55	23.8%	176	76.2%	0.736
African-Brazilian	246	15	6.1%	3	20.0%	12	80.0%	
**Comorbidities ^1^**								
No	252	216	85.7%	51	23.6%	165	76.4%	0.589
Yes	252	36	14.3%	10	27.8%	26	72.2%	
**Immunosuppression**								
No	252	241	95.6%	58	24.1%	183	75.9%	0.808
Yes	252	11	4.4%	3	27.3%	8	72.7%	

Available comorbidities: asthma, kidney disease, liver disease, hematologic diseases, seizures, cancer, immunosuppression, genetic syndrome.

## Data Availability

Anonymized data are available from the authors upon request.
